# Phase 1 dose-escalation study of momelotinib, a Janus kinase 1/2 inhibitor, combined with gemcitabine and nab-paclitaxel in patients with previously untreated metastatic pancreatic ductal adenocarcinoma

**DOI:** 10.1007/s10637-018-0650-5

**Published:** 2018-07-30

**Authors:** Kimmie Ng, Andrew Hendifar, Alexander Starodub, Jorge Chaves, Yingsi Yang, Brian Koh, David Barbie, William C. Hahn, Charles S. Fuchs

**Affiliations:** 1 Dana-Farber Cancer Institute, Harvard Medical School, Boston, MA USA; 2 Cedars-Sinai Samuel Oschin Comprehensive Cancer Institute, Los Angeles, CA USA; 3 Parkview Physicians Group Medical Oncology, Fort Wayne, IN USA; 4 Northwest Medical Specialties, Tacoma, WA USA; 5 Gilead Sciences, Foster City, CA USA; 6 Yale Cancer Center/Smilow Cancer Hospital, New Haven, CT USA

**Keywords:** Momelotinib, JAK inhibitor, TBK1 inhibitor, Phase 1

## Abstract

*Purpose* Preclinical evidence suggests the importance of Janus activating kinase (JAK) and TANK-binding kinase 1 (TBK1) in pancreatic ductal adenocarcinoma (PDAC). We evaluated the safety and efficacy of momelotinib (MMB), a JAK1/2 inhibitor with additional activity against TBK1, plus albumin-bound paclitaxel + gemcitabine (nab-P + G), in patients with previously untreated metastatic PDAC. *Experimental Design* Patients were enrolled into five cohorts of increasing doses of MMB between 100 and 200 mg administered once or twice daily in combination with nab-P + G in 28-day cycles to determine maximum tolerated dose (MTD). Safety, efficacy, pharmacokinetics, and pharmacodynamics were assessed for all patients. *Results* Twenty-five patients were enrolled. Dose-limiting toxicities of Grade 3 diarrhea occurred in 1 patient each in the 100 and 200 mg MMB once-daily dose groups. MTD was not reached. The 200 mg MMB twice-daily was the maximum administered dose. Objective response rate was 28% (all partial responses), and 13 (52%) patients had a best response of stable disease. The most common adverse events (AEs) were fatigue (80%), nausea (76%), and anemia (68%). Grade 3 or 4 AEs, most commonly neutropenia (32%), were reported by 88% of patients, of which 44% were considered related to MMB. Pharmacokinetic analyses showed MMB concentrations were too low for TBK1 inhibition. *Conclusions* MMB was safe and well tolerated in combination with nab-P + G. As no OS or PFS benefit vs nab-P + G was apparent in context of suboptimal engagement of the target TBK1, this study does not support further development of MMB as a first-line therapy in pancreatic cancer.

## Introduction

As the third leading cause of cancer-related death in the United States, estimates for 2018 are that more than 55,440 people will be diagnosed and nearly 44,330 will die from pancreatic cancer [1]. Pancreatic cancer has an exceedingly poor prognosis, with a majority of patients dying within a year of diagnosis and only 9% surviving past 5 years [1]. Consequently, new and effective therapies are urgently needed.

Pancreatic ductal adenocarcinoma (PDAC), the most common pancreatic cancer, is often detected at a late stage, precluding potentially curable surgical intervention. Approved chemotherapies offer a relatively modest survival benefit of 4–5 months at best [2].

*KRAS* mutations are present in >90% of pancreatic carcinomas, and initiate and drive aggressive tumor growth by engaging multiple downstream signaling pathways [2]. Efforts to inhibit *KRAS* directly have remained elusive, and strategies to target the downstream PI3K/Akt and MAP kinase pathways have been disappointing [3–5]. TANK-binding kinase 1 (TBK1), which regulates innate immunity, lies downstream of *KRAS*. Activated TBK1 drives specific NF-κB–regulated survival signals that promote continued proliferation and/or survival and feed forward in an autocrine cytokine circuit through Janus activating kinase (JAK)/STAT signaling [6–8]. Aberrant activation of STAT3 is required for PDAC initiation and proliferation [9]. Suppression of TBK1 induces apoptosis of *KRAS*-driven cancer cells, and JAK2 inhibition of STAT3 activation results in decreased growth [9, 10]. Consequently, JAK2 and TBK1 inhibitors may have utility in this cancer.

Momelotinib (MMB) is an investigational oral agent with potent JAK1/2 inhibitory activity that also inhibits TBK1 [11]. The objective of this study (ClinicalTrials.gov Identifier: NCT02101021) was to evaluate the safety, efficacy, and pharmacokinetics of MMB combined with albumin-bound paclitaxel + gemcitabine (nab-P + G), and define the maximum tolerated dose (MTD). Enrollment was stopped early at the inflection point between the phase 1 and planned phase 3 portions of the study due to limited efficacy and suboptimal TBK1 pharmacodynamics. We report the final results of the trial.

## Methods

### Patient population

Patients were ≥ 18 years of age with previously untreated metastatic PDAC (measurable per Response Evaluation Criteria in Solid Tumors [RECIST] v 1.1); and an Eastern Cooperative Oncology Group (ECOG) performance status ≤1 and adequate hematologic, renal, and hepatic function. Patients were excluded for any current or previous treatment with biologic, small-molecule, immunotherapy, chemotherapy, or other agents for metastatic pancreatic carcinoma; uncontrolled intercurrent illness; recent major or minor surgery; certain prior or secondary malignancies; human immunodeficiency virus infection; hepatitis A, B, or C infection; peripheral neuropathy ≥Grade 2; central nervous system metastases; histology other than pancreatic adenocarcinoma; external biliary drain; myocardial infarction or unstable/uncontrolled cardiac disease; uncontrolled hypertension; and use of strong CYP3A4 inducers within 2 weeks prior to first dose of study medication. All patients provided written informed consent prior to enrollment.

### Trial design and treatments

The study was conducted at four sites in the United States from June 2014 to April 2017 in accordance with the Declaration of Helsinki, Good Clinical Practice guidelines, and relevant regulatory laws. Each center’s institutional review board approved the study protocol. All patients provided written informed consent.

Screening occurred within 21 days before the first MMB dose and included medical history and prior/concomitant medication review, physical exam and assessments of vital signs, ECOG performance status, pregnancy test (females), laboratory assessment, adverse event (AE) assessment, and staging CT or MRI. MMB was given orally once or twice daily as per the assigned dosing cohort: Cohort 1–100 mg MMB once daily; Cohort 2–150 mg MMB once daily; Cohort 3–200 mg MMB once daily; Cohort 4–150 mg MMB twice daily; and Cohort 5–200 mg MMB twice daily. All patients received nab-P + G intravenously (125 mg/m^2^ nab-paclitaxel followed by 1000 mg/m^2^ G as per institutional standard of care) on Days 1, 8, and 15 of each 28-day treatment cycle. Each dose cohort consisted of 3 patients, unless a dose-limiting toxicity (DLT) was experienced during the first 28 days of treatment. If one DLT was experienced, 3 additional patients were enrolled in the dose cohort. If a second DLT was experienced in the same cohort, MTD was considered to be exceeded. Dose escalation continued from Dose Level 2 to 3 to 4 to 5 if no DLT occurred in 3 evaluable subjects or < 2 DLTs occurred in 6 evaluable patients within each dose level.

Staging CT or MRI scans were performed at baseline and approximately every 8 weeks following Cycle 1 Day 1. Imaging modalities were maintained over the course of the study. Treatment was to continue in the absence of disease progression, unacceptable toxicity, consent withdrawal, or refusal of treatment. After discontinuation of treatment, patients were followed for safety for 30 days.

Primary endpoint was incidence of DLT(s) based on the Common Terminology Criteria for Adverse Events (CTCAE) version 4.03. Secondary endpoints were overall survival (OS) (defined as the time interval from the first dose of MMB to death from any cause; patients lost to follow-up or who survived until the end of study were censored at the last date that they were known to be alive), progression-free survival (PFS) (defined as the time interval from the first dose of MMB to the earlier of the first documentation of definitive disease progression or death from any cause), overall response rate (ORR) (proportion of patients who achieved a complete or partial response [CR or PR, respectively] during MMB therapy), and duration of response (time from CR or PR to definitive disease progression or death from any cause).

### Pharmacokinetic analyses

Blood samples were collected from patients prior to the MMB dose and at specified time points up to 24 h post-dose on Cycle 1 Day 15. Pharmacokinetic (PK) parameters were estimated using Phoenix WinNonlin^®^ software (Certara USA, Inc., Princeton, NJ) using standard noncompartmental methods.

### Statistical analysis

Planned enrollment was up to 30 patients based on the standard 3 + 3 design. The efficacy analyses were conducted in all enrolled patients. Safety analyses were conducted in all enrolled patients who received ≥1 dose of study treatment. Descriptive summary statistics were provided for patient characteristics and safety variables. Kaplan-Meier estimates were used for OS, PFS, duration of response, and their 95% confidence intervals (CIs). The ORR was summarized for each dose level cohort with corresponding 2-sided 95% exact CIs based on the Clopper-Pearson method.

## Results

**Fig. 1:**
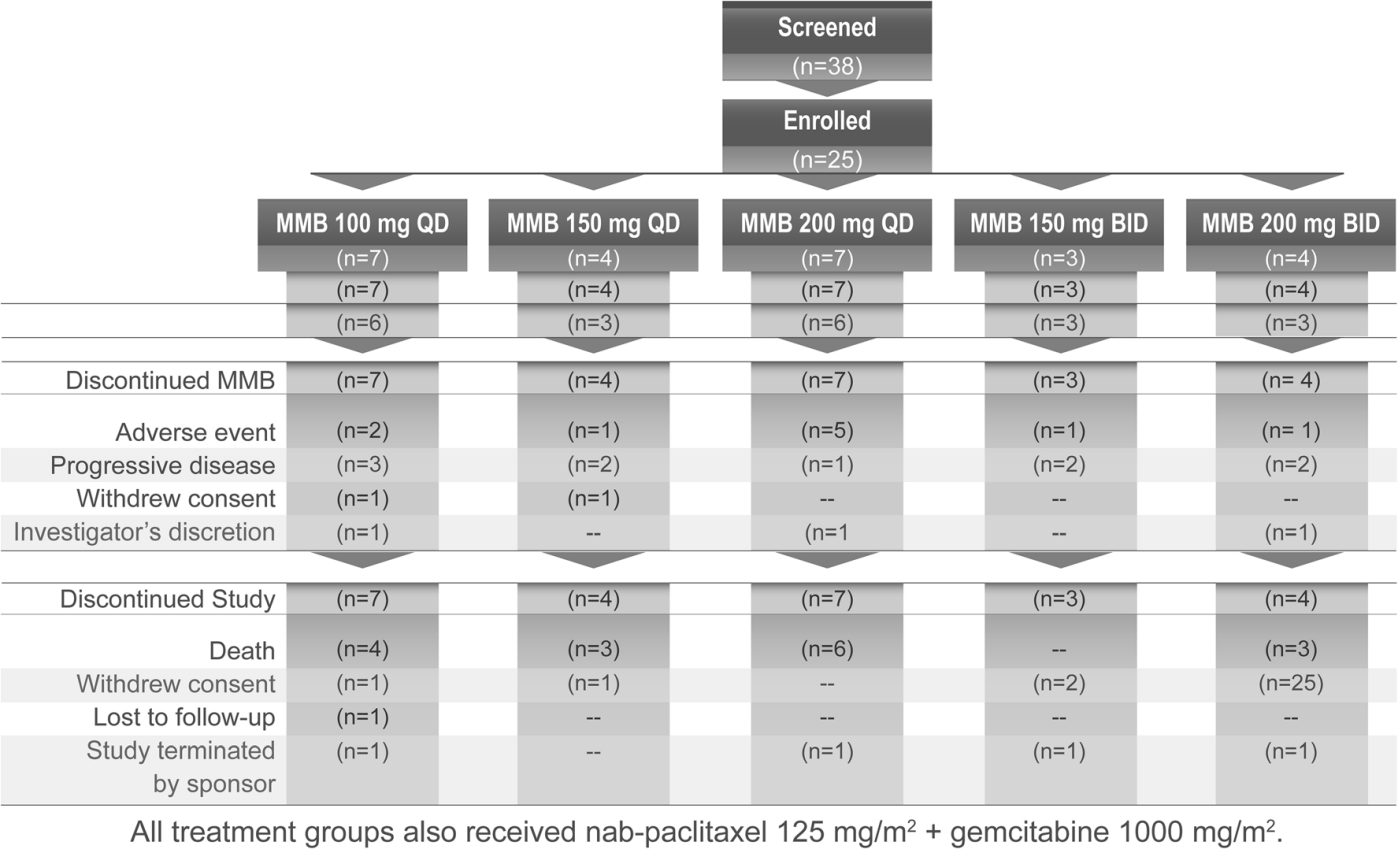
Patient disposition. BID, twice daily; MMB, momelotinib; QD, once daily

The study screened 38 patients and enrolled 25 patients with a mean age 60.7 ± 10.2 years and mean body mass index 25.8 ± 5.4 kg/m^2^; most were male (68%) and white (88%). Patient disposition is shown in Fig. 1. Mean duration of MMB exposure was 22.2 ± 15.3 weeks. Mean number of cycles was 5.6 ± 3.8. All 25 patients discontinued MMB nab-P + G treatment, most commonly for disease progression and AEs. Patients discontinued the study due to death (*n* = 16 [64.0%]), withdrawal of consent (*n* = 4 [16.0%]), study termination by the sponsor (n = 4 [16.0%]), or loss to follow-up (n = 1 [4.0%]).

### Safety

Two DLTs occurred. The first DLT was Grade 3 diarrhea with intermittent fever in a 68-year-old male with multiple chronic concomitant illnesses in the 100 mg once-daily dose group. The second patient was a 62-year-old male with multiple chronic concomitant illnesses in the 200 mg once-daily group who experienced Grade 3 diarrhea beginning on Cycle 1 Day 18 and was admitted to the hospital for Grade 3 febrile neutropenia on Cycle 1 Day 21.

MTD was not reached. The 200 mg MMB twice-daily dose was the maximum administered dose (MAD).

All patients experienced at least one treatment-emergent AE (TEAE). The most common TEAEs were fatigue (80%); nausea (76%); anemia (68%); diarrhea (64%); pyrexia (56%); constipation and vomiting (52% each); peripheral edema (48%); abdominal pain (44%); alopecia, decreased appetite, and dysgeusia (40% each); and hypertension, peripheral neuropathy, and peripheral sensory neuropathy (36% each). Serious AEs (SAEs) were experienced by 72% of patients (24% considered related to MMB). 88% of patients reported Grade 3 or 4 TEAEs, most commonly neutropenia (Grades 3 and 4, 16% each) and anemia and pneumonia (all Grade 3, 24% each). SAEs were considered related to MMB in 24% of patients. Grade 3 MMB-related AEs included diarrhea (12.0%); peripheral sensory motor neuropathy, anemia, thrombocytopenia, and decreased neutrophil count (8.0% each); and fatigue, peripheral edema, malaise, generalized edema, polyneuropathy, tremor, febrile neutropenia, dehydration, cachexia, decreased weight, respiratory distress, hypertension, deep vein thrombosis, and nephrolithiasis (4.0% each). Grade 4 events included embolic stroke, neutropenia, and increased blood uric acid (4.0% each). There were no deaths due to TEAEs.

Peripheral neuropathy, a predefined AE of interest, occurred in 15 patients (60%), with a higher incidence at the two highest dose levels, and led to discontinuation of MMB in 2 patients (8%, Grade 3 peripheral sensory motor neuropathy and Grade 3 mixed polyneuropathy). Investigators attributed peripheral sensory neuropathy and peripheral neuropathy as being related to MMB in 6 (24%) and 4 (16%) patients, respectively; related to nab-paclitaxel in 9 (36%) and 8 (32%) patients, respectively; and related to gemcitabine in 7 (28%) and 5 (20%) patients, respectively.

### Efficacy

**Fig. 2:**
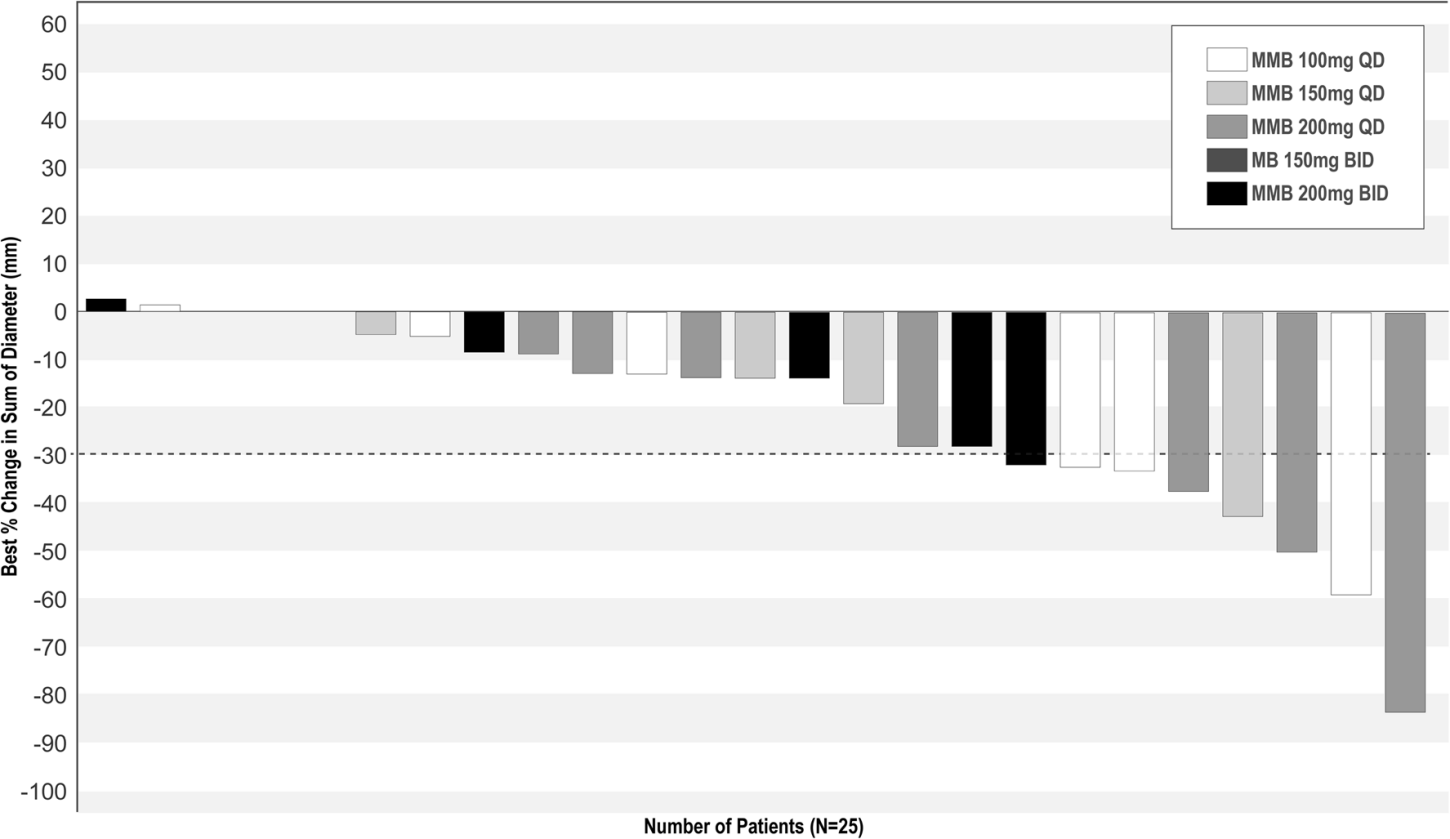
Waterfall plot of best percentage change from baseline in tumor size by dose level. BID, twice daily; MMB, momelotinib; QD, once daily

Response rates are shown in Table 1. The best percentage change from baseline in tumor size is shown in Fig. 2. There were no CRs and 7 PRs across the dose cohorts. Thus, the ORR in the overall study population was 28% (95% CI: 12.1%–49.4%). A total of 13 patients (52%) had stable disease as the best response, including all 4 patients in the 200 mg twice-daily cohort (MAD). For the overall study population, the median (95% CI) OS was 8.7 (6.7, 18.8) months and the median (95% CI) PFS was 5.7 (5.3, 7.2) months. The median (95% CI) duration of response for the 7 patients with a PR was 4.0 (0.5, not reached) months.

**Table 1:** Best Overall Response n, (%)

n, (%)	100 mg MMB QD n = 7	150 mg MMB QD n = 4	200 mg MMB QD n = 7	150 mg MMB BID n = 3	200 mg MMB BID n = 4
ORR (CR + PR)	2 (28.6)	1 (25.0)	3 (42.9)	1 (33.3)	0
CR	0	0	0	0	0
PR	2 (28.6)	1 (25.0)	3 (42.9)	1 (33.3)	0
SD	2 (28.6)	2 (50.0)	4 (57.1)	1 (33.3)	4 (100.0)
PD	1 (14.3)	1 (25.0)	0	0	0
Not evaluable	2 (28.6)	0	0	1 (33.3)	0

BID, twice daily; CR, complete response; MMB, momelotinib; ORR, overall response rate; PR, partial response; PD, progressive disease; QD, once daily; SD, stable disease

### Pharmacokinetic assessment

Twenty subjects were included in the PK analyses. Of the 5 patients not included in the PK analyses, 4 had MMB dosing interrupted or decreased at the time of PK sampling and 1 had discontinued the study before PK samples were taken. PK parameters for MMB and its major metabolite, GS-644603, are shown in Table 2. On Cycle 1 Day 15, plasma concentrations of MMB reached a peak approximately 3–4.5 h after the first dose of MMB; median terminal half-life was approximately 5–6.6 h. Increases in exposure of MMB were less than dose-proportional; peak plasma concentration (C_max_) and area under the curve (AUC_tau_) were less than 2-fold higher after the 200 mg once-daily dose of MMB compared with the 100 mg once-daily dose. Based on the C_max_ of approximately 300 ng/mL, we estimate transient maximal TBK1 inhibition in the 70% maximal effective concentration (EC_70_) range.

**Table 2:** Steady-state MMB PK Parameters

Parameter^a^	100 mg MMB QD n = 6	150 mg MMB QD n = 3	200 mg MMB QD n = 6	150 mg MMB BID n = 1^b^	200 mg MMB BID n = 3
Momelotinib					
C_max_, ng/mL	257.8 (83.4)	200.3 (19.0)	363.8 (92.3)	215.0	330.0 (56.2)
AUC_tau_, h•ng/mL	2004.5 (64.1)	1679.6 (53.7)	3340.1 (101.3)	1518.2	1976.8 (54.4)
T_max_, hr	4.5 (2.0, 6.0)	3.2 (2.1, 4.0)	3.5 (1.0, 7.7)	3.0	3.0 (1.0, 4.0)
T _1/2_, hr	5.4 (5.2, 5.5)	6.6 (5.0, 8.1)	5.3 (4.0, 6.8)	5.0	5.5 (3.8, 5.9)
GS-644603					
C_max_, ng/mL	170.4 (51.3)	513.7 (53.2)	342.7 (52.1)	247.0	349.7 (41.7)
AUC_tau_, h•ng/mL	2132.5 (67.2)	5431.4 (50.8)	3772.5 (46.3)	1922.9	2693.4 (25.7)
T_max_, hr	5.0 (2.0, 6.0)	4.0 (3.1, 6.0)	3.8 (3.0, 8.0)	2.97	3.0 (1.0, 4.0)
T _1/2_, hr	9.1 (6.4, 10.0)	6.3 (5.7, 6.9)	6.8 (4.1, 8.0)	6.620	28.4 (4.3, 31.7)
GS-644603/momelotinib ratio					
AUC_tau_	1.2 (67.5)	4.6 (93.6)	1.5 (38.7)	1.2	1.7 (65.0)
C_max_	0.9 (80.0)	2.7 (67.9)	1.5 (67.0)	1.1	1.4 (65.2)

^a^Data for C_max_ and AUC_tau_ are presented as the mean (% coefficient of variation); data for T_max_ and t_1/2_ are presented as median (first quarter, third quarter); and GS-644603/momelotinib ratios are presented as the mean (% coefficient of variation)

^b^Standard deviation was not calculated because n = 1 for this parameter at the given dose level

AUC_tau_, area under the curve tau; BID, twice daily; C_max_, peak plasma concentration; MMB, momelotinib; QD, once daily; t_max_, amount of time that a drug is present at the maximum concentration in serum

On Cycle 1 Day 15, plasma concentrations of GS-644603 reached a peak approximately 3–5 h after the first dose of MMB. Across dose levels, the median terminal half-life of GS-644603 was approximately 6–28 h. Mean metabolite-to-parent ratio across dosing groups ranged from 0.9 to 2.7 and 1.2 to 4.6 for C_max_ and AUC_tau_, respectively.

## Discussion

In this study, MMB in doses of up to 200 mg twice daily (MAD) was safe in combination with nab-P + G. Only 2 DLTs of Grade 3 diarrhea were experienced. These were insufficient to guide determination of the MTD in this patient population.

OS and PFS values varied inconsistently with dose, but were generally comparable to those observed with nab-P + G alone in the MPACT trial (median OS = 8.5 months in the nab-P + G group [12]. There was no apparent dose relationship in terms of tumor response, with no CRs in any of the dose cohorts, the highest ORR in the 200 mg once-daily cohort (middle dose), and an ORR of 0 in the cohort receiving the MAD. This is consistent with the lack of dose-proportional PK increases that were observed.

The limited efficacy demonstrated and lack of apparent relationship between dose and efficacy at the inflection point between the dose-finding phase 1 portion of the trial and the randomized phase 3 portion of the trial led to a decision not to initiate the planned phase 3 portion of the trial. Announcement of the results of a trial assessing the combination of ruxolitinib, another JAK1/2 inhibitor, with capecitabine in patients with metastatic pancreatic cancer that was refractory to gemcitabine occurred as the phase 1 portion of this study was concluding, and further supported the decision to not advance to the phase 3 portion. That trial found that although the prespecified primary endpoint of OS was not met in the overall study population (HR for OS comparing ruxolitinib vs placebo = 0.79 [95% CI, 0.53–1.18]; *P* = 0.25), a subgroup analysis suggested benefit for patients with C-reactive peptide >13 mg/L (HR for OS = 0.47 [95% CI, 0.26–0.85], *P* = 0.011) [13]. Two phase 3 trials (JANUS-1 and -2) were initiated to confirm these initial findings. Unfortunately, interim analyses of both the JANUS-1 and JANUS-2 trials resulted in early termination for futility (HR for ruxolitinib vs placebo for OS = 0.969 [95% CI, 0.75–1.26], *P* = 0.409; and HR for OS = 1.584 [95% CI, 0.89–2.83], *P* = 0.942, respectively) [14].

No unexpected toxicities occurred with MMB. TEAEs were consistent with those reported previously in a myelofibrosis population [15, 16]. AEs consistent with JAK1/2 inhibition are anemia, thrombocytopenia, and neutropenia, which were observed in 68%, 20%, and 32% of patients, respectively, in this study. These AEs have also been observed in trials conducted in patients with myelofibrosis treated with MMB [15, 16] and with ruxolitinib [17, 18]. Peripheral neuropathy is known to be associated with JAK inhibition, and resulted in discontinuation of MMB in 2 patients (8%) in this study. Assessment of this AE is confounded by the fact that nab-paclitaxel is also associated with neuropathy, which may explain the higher incidence of neuropathy with MMB in PDAC patients (60%) than in myelofibrosis patients (10–44%) [17–19].

PK analysis was generally comparable to prior evaluations [16, 20]. Maximum momelotinib plasma concentrations were approximately 300 ng/mL (equal to 0.72 μmol/L). Our estimates of transient maximal TBK1 inhibition in the EC_70_ range are insufficient to achieve clinically meaningful levels of TBK1 inhibition [21]. Tonic inhibition at significantly higher EC levels would theoretically be required for therapeutic efficacy.

## Conclusion

Momelotinib is an investigational agent which is well tolerated in combination with gemcitabine and nab-paclitaxel for previously untreated PDAC. This study was stopped prior to entering the planned phase 3 portion of the trial because of limited efficacy in context of suboptimal dose/exposure of momelotinib for meaningful engagement of the target TBK1 and a similar lack of efficacy of JAK inhibition in other trials. The disappointing results in this study do not support further development of MMB as a component of first-line therapy in pancreatic cancer, but TBK1 remains a significant target of interest.
